# Adaptive Self-Attention Graph Pooling for Drug–Target Affinity Prediction

**DOI:** 10.3390/ijms27135861

**Published:** 2026-06-29

**Authors:** Changli Li, Guangyue Li

**Affiliations:** School of Artificial Intelligence, Nanjing University of Information Science & Technology, Nanjing 210044, China; 202212620007@nuist.edu.cn

**Keywords:** drug–target affinity, graph neural networks, adaptive graph pooling, self-attention, Transformer

## Abstract

Drug–target affinity (DTA) prediction is a critical step in drug discovery and precision medicine. Although graph neural networks (GNNs) have achieved remarkable progress, existing graph pooling methods rely on fixed ratios, failing to adapt to the structural diversity of molecules and proteins, which leads to information loss or redundant feature retention. To address this issue, we propose the Adaptive Self-Attention Graph Pooling (ASAGPooling) mechanism, which introduces a learnable pooling ratio that dynamically adjusts node retention during training. Furthermore, we develop ASAG-DTA, a multi-modal framework that integrates GNNs with Transformers to jointly model molecular graphs, protein contact maps, SMILES sequences, and FASTA sequences. While ASAGPooling achieves competitive prediction accuracy (MSE = 0.186 on Davis), we acknowledge that it does not surpass the state-of-the-art DynHeter-DTA (MSE = 0.130), which incorporates a more complex dynamic heterogeneous graph architecture. Instead, the key contribution of ASAGPooling lies in its adaptability, interpretability, and computational efficiency. It can eliminate the need for manually tuned pooling ratios, enable direct visualization of retained key residues/atoms, and reduce model complexity. This makes ASAG-DTA a practical lightweight alternative for large-scale virtual screening scenarios where computational resources are constrained.

## 1. Introduction

Quantifying drug–target affinity (DTA) is a fundamental task in drug discovery and precision medicine, directly informing therapeutic efficacy and safety assessments of candidate compounds [1,2]. While experimental techniques such as isothermal titration calorimetry (ITC) and surface plasmon resonance (SPR) provide accurate measurements, they are time-consuming, resource-intensive, and ill-suited for high-throughput screening [3]. Consequently, developing efficient and accurate computational methods for DTA prediction has become an urgent priority.

### 1.1. From Traditional Methods to Deep Learning

Existing computational approaches for drug–target affinity prediction can be broadly categorized into three types: structure-based simulations, conventional machine learning (ML), and deep learning [4]. Structure-based methods, including molecular docking [5] and molecular dynamics simulations [6], depend on high-resolution three-dimensional structures and entail substantial computational costs. Early ML approaches [7,8,9], such as KronRLS [10] and SimBoost [11], derive features from protein and compound sequences but rely heavily on manual feature engineering, which constrains their generalizability across diverse datasets.

Deep learning has recently revolutionized DTA prediction [12,13,14]. Models like DeepDTA [15] and WideDTA [16] use convolutional neural networks (CNNs) to encode drug SMILES and protein FASTA sequences, while TransformerCPI [17] adopts the Transformer architecture [18]. However, sequence-only methods suffer from two intrinsic limitations: (1) they require padding/truncation to uniform lengths, discarding information, and (2) they cannot capture critical spatial relationships that emerge only in folded 3D structures.

### 1.2. Graph Neural Networks and the Pooling Bottleneck

To overcome these limitations, graph neural networks (GNNs) have been widely adopted in drug discovery [19,20,21], as both drugs and proteins can be naturally represented as graphs. GraphDTA [22] constructs molecular graphs with atoms as nodes and bonds as edges, while DGraphDTA [23] builds residue contact graphs for proteins. Despite their success, existing GNN-based DTA models face a critical bottleneck: how to perform graph pooling effectively.

Global pooling is computationally efficient but loses local structure. Hierarchical pooling methods, while reducing complexity, share a common flaw—the fixed pooling ratio. Methods like TopKPooling [24], DiffPool [25], and even the attention-based SAGPool [26] all retain or discard nodes based on a predetermined, static proportion. This is suboptimal for molecular and protein graphs, where the distribution of functionally critical regions varies dramatically: a small drug molecule may need to retain 80% of its nodes to preserve key functional groups, while a large protein contact graph may effectively compress to 30% of its residues. Fixed ratios thus inevitably cause information loss in compact molecules or redundant feature retention in large proteins.

### 1.3. Research Gap and Proposed Solution

To date, no adaptive graph pooling method with a learnable retention ratio has been developed for DTA prediction. This paper fills that gap by proposing the adaptive self-attention graph pooling (ASAGPooling) mechanism. ASAGPooling introduces a learnable pooling ratio, optimized via gradient descent, which dynamically adjusts node retention during training. This adaptively preserves structurally critical regions while reducing computational redundancy. Building on this mechanism, we construct ASAG-DTA, a multimodal deep learning framework that integrates:

- Drug molecular graphs processed by Graph Attention Networks (GATv2);
- Drug SMILES sequences processed by Transformer;
- Protein residue contact graphs processed by Graph Isomorphism Networks (GINs);
- Protein FASTA sequences processed by Transformer;
- Extended Connectivity Fingerprints (ECFPs) for global drug features.

### 1.4. Recent AI Advances in Structural Biology and Bioinformatics

Our work is situated within a broader context of rapid AI advances in biology. Notably, AlphaFold [27] demonstrated that deep learning can predict protein structures with atomic accuracy, fundamentally transforming structure-based drug design. Recent scientometric analyses have further confirmed that AlphaFold is driving an astonishing development trend in molecular biology and drug discovery, with “structure prediction,” “artificial intelligence,” and “molecular dynamics” identified as core research hotspots [28]. Transformer-based protein language models, such as ESM-1b [29] and ProtTrans [30], have shown that pre-training on massive sequence corpora captures rich evolutionary and structural patterns without explicit structural input. Comprehensive reviews have systematically surveyed machine learning applications in drug–target interaction [31], deep learning for binding prediction [32], and AI resources for drug discovery [33]. These advances provide both inspiration and complementary tools: AlphaFold-predicted structures could enhance protein graph construction, while transformer-based protein embeddings could serve as stronger sequence encoders. Our ASAG-DTA framework, with its adaptive pooling mechanism, offers a new perspective on extending learnable graph pooling to bioinformatics tasks.

### 1.5. Contributions

The key contributions of this study are summarized as follows:

1. Adaptive graph pooling (ASAGPooling): A novel self-attention-based pooling method with a learnable ratio optimized via gradient descent, enabling dynamic node selection that preserves critical information across diverse molecular graphs.
2. Multimodal ASAG-DTA framework: Integration of GNNs (GATv2, GIN), Transformers (for SMILES and FASTA sequences), and ECFP within a unified architecture.
3. Competitive Performance: Extensive experiments on Davis [34] and KIBA [35] benchmarks demonstrate that ASAG-DTA achieves favorable results across multiple evaluation metrics, including MSE, CI, and $r_{m}^{2}$.
4. Comprehensive ablation and analysis: Detailed ablation studies validate each component’s contribution, and visualization analyses illustrate how ASAGPooling preserves key structural regions while reducing redundancy.

## 2. Results

### 2.1. Datasets and Evaluation Protocol

To assess the performance of our proposed ASAG-DTA model, we conducted experiments on two publicly available benchmark datasets widely used for DTA prediction: the Davis dataset [34], which contains 30,056 documented drug–protein binding pairs, and the KIBA dataset [35], comprising 118,254 interaction records. Table 1 presents the key statistical information for both datasets.

In accordance with standard evaluation protocols, we partitioned each dataset into training and test sets using a 5:1 ratio, and further applied five-fold cross-validation on the training portion. For quantitative evaluation, we employed three complementary metrics: mean squared error (MSE) to measure prediction accuracy, Concordance Index (CI) to assess ranking consistency, and the relative coefficient of determination ($r_{m}^{2}$) as a robust indicator of explained variance.

### 2.2. Comparison with Baseline Methods

We compare ASAG-DTA with nine baseline methods, including traditional (KronRLS, SimBoost), deep learning (DeepDTA, WideDTA), and GNN-based (GraphDTA, MGraphDTA, DGraphDTA) approaches.

Davis Dataset. Table 2 presents the comparison. ASAG-DTA achieves superior results: MSE = 0.186, CI = 0.915, and $r_{m}^{2}=0.765$. Compared to the best baseline DGraphDTA (MSE = 0.202, CI = 0.904, $r_{m}^{2}=0.700$), ASAG-DTA improves MSE by 7.9%, CI by 1.2% and $r_{m}^{2}$ by 9.3%.

KIBA Dataset. Table 3 shows similar improvements. ASAG-DTA achieves MSE = 0.128, CI = 0.906, and $r_{m}^{2}=0.798$. While DGraphDTA reports a slightly lower MSE (0.126), ASAG-DTA achieves superior CI (0.906 vs. 0.904) and $r_{m}^{2}$ (0.798 vs. 0.786), indicating better ranking consistency and explained variance.

### 2.3. Binding Affinity Prediction on Testing Data

To evaluate the predictive reliability of our model, we compared the estimated binding affinities against the ground-truth values for the test sets of both the Davis and KIBA datasets. A strong positive correlation was observed between the predicted and actual measurements, with data points in the scatter plots clustering tightly around the diagonal line representing perfect prediction. This close agreement demonstrates that our model achieves accurate and stable fitting of the underlying data distribution.

To systematically identify and characterize prediction errors, we examined data points whose absolute residuals fell within the top 5% of the error distribution for each dataset. The mean absolute error (MAE) across all test samples was 0.178 for Davis and 0.159 for KIBA. In contrast, the outlier subset—comprising approximately 5.01% of all samples—exhibited substantially larger MAE values of 1.233 and 0.1124, respectively. These results confirm that the 95th percentile threshold effectively isolates a high-error tail that deviates markedly from the overall population mean.

When we grouped the outlier samples by their associated compounds versus proteins, a clear pattern emerged: the majority of anomalies originated from the compound side rather than from the protein side. Several specific compounds—namely 11,409,972, 11,984,591, 16,038,120, and 126,565—showed average prediction errors exceeding 1.0 across multiple target proteins. For instance, compound 16,038,120 produced large deviations not only for AK (2.14) but also for BMPR1B (1.82), TSSK1B (1.80), TRKB (1.57), and CHEK1 (1.49). Similarly, compound 11,409,972 yielded high errors for EPHB6 (2.31), MET (1.90), LOK (1.66), and TNK1 (1.65). These observations suggest that such compounds are not merely difficult for a single target; rather, they exhibit persistently large prediction errors across multiple unrelated targets.

To better understand the root causes of these systematic errors, we performed an in-depth analysis of the structural and physicochemical properties of the problematic compounds. The results revealed that their core scaffolds and substructures are highly diverse, as indicated by low pairwise Tanimoto similarity scores, ruling out the possibility that they belong to a single chemical series that could introduce systematic bias. However, at the level of global physicochemical characteristics, these compounds share several common traits: high molecular weights (exceeding 500 Da), elevated LogP values (greater than four), and the presence of multiple aromatic rings along with numerous hydrogen-bond donor and acceptor sites. Taken together, these features characterize the outlier compounds as “complex, lipophilic, and high-molecular-weight” molecules.

### 2.4. Ablation Study

To identify the factors that most significantly influence our model’s predictive performance, we carried out a series of ablation experiments using different architectural variants. The specific configurations we compared are enumerated below:

1. Configuration A: Without a heterogeneous graph, using GCN instead of GIN and SAGPooling;
2. Configuration B: Without a heterogeneous graph, using GIN and SAGPooling;
3. Configuration C: Using a dynamic heterogeneous graph with GIN and SAGPooling.

The results of these ablation experiments revealed that two factors are primarily responsible for the model’s predictive strength: the incorporation of a heterogeneous graph structure, and the joint application of GIN with SAGPooling. Across both the Davis and KIBA datasets, the poorest performance was observed for Configuration A, which omitted the heterogeneous graph and replaced GIN and SAGPooling with a standard GCN. Substituting GIN and SAGPooling while still omitting the heterogeneous graph (Configuration B) led to noticeable improvements. Notably, the best results across all evaluation metrics were attained by Configuration C, which employed the dynamic heterogeneous graph together with GIN and SAGPooling. These findings confirm that introducing the dynamic heterogeneous graph, along with the synergistic combination of GIN and SAGPooling, substantially boosts predictive capability, underscoring the essential role these components play in enhancing model performance.

We further conducted ablation experiments to quantify the individual contribution of each module within the complete ASAG-DTA framework:

- w/o ASAG: Replace ASAGPooling with global mean pooling (no hierarchical pooling).
- w/o Adaptive: Use SAGPooling with fixed ratio (k=0.5) instead of ASAGPooling.
- w/o Drug Seq: Remove Transformer encoder for drug SMILES sequences.
- w/o Protein Seq: Remove Transformer encoder for protein FASTA sequences.
- w/o Fingerprint: Remove ECFP features.
- ASAG-DTA (Full): All components included.

Table 4 presents the ablation results on the Davis dataset.

Key observations:

1. ASAGPooling vs. global pooling: The complete model significantly outperforms the global pooling variant (MSE: 0.186 vs. 0.215, improvement of 13.5%), demonstrating the importance of hierarchical pooling for preserving critical structural information.
2. Adaptive vs. fixed ratio: The adaptive mechanism (ASAGPooling) outperforms the fixed ratio SAGPooling (MSE: 0.186 vs. 0.198), confirming that learning the pooling ratio from the data is beneficial.
3. Transformer contributions: Both drug and protein sequence encoders contribute meaningfully. Removing drug sequence features increases MSE to 0.208; removing protein sequence features increases MSE to 0.202.
4. Fingerprint contribution: ECFP features provide complementary global information, with removal increasing MSE to 0.195.

Table 5 and Table 6 show additional ablation results for the DynHeter-DTA model (the predecessor of ASAG-DTA).

The baseline configurations (’Without heterogeneous graph…’) and their results are consistent with those reported in our previous study [36]. We re-evaluated these configurations under the same random seed and data split to ensure comparability with ASAG-DTA.

### 2.5. Analysis of Adaptive Pooling Ratios

To understand how ASAGPooling adapts to different inputs, we analyze the learned pooling ratios across the Davis dataset.

Key findings:

- Drug graphs: Learned ratios average 0.62 (range: 0.45–0.78). Smaller drug molecules (<30 atoms) tend to have higher retention ratios (>0.70), which preserve most atoms. Larger drug molecules (>80 atoms) have lower retention ratios (∼0.55), selectively retaining key functional groups.
- Protein graphs: Learned ratios average 0.41 (range: 0.28–0.55). Large proteins (>500 residues) show lower retention ratios (∼0.35), efficiently pruning non-interacting surface residues.
- Convergence: The adaptive parameter α converges within 500–1000 epochs, stabilizing at near-optimal values without explicit regularization.

This adaptive behavior confirms that ASAGPooling successfully captures structural heterogeneity, preserving more nodes from information-dense small graphs while aggressively pruning larger graphs.

### 2.6. Cold-Start Evaluation

To assess generalization to unseen entities, we conducted three cold-start scenarios on the Davis dataset:

- Cold drug: Test drugs not seen during training (20% of drugs held out).
- Cold protein: Test proteins not seen during training (20% of proteins held out).
- Cold pair: Both drug and protein unseen (drug–protein pairs where neither entity appears in training).

Table 7 presents the results. As expected, performance decreases from the random split setting, with cold pair being the most challenging. Notably, ASAG-DTA maintains reasonable performance (CI > 0.85) in all scenarios, demonstrating its ability to leverage similarity information for unseen entities.

### 2.7. Visual Explanation

#### 2.7.1. Visualization of the Dynamic Heterogeneous Graph

To gain insight into how our variable heterogeneous graph prediction model operates, we performed a visual analysis of the constructed drug–protein heterogeneous graph. In conventional static graph formulations, edge connections are typically determined by predefined similarity measures or fixed interaction strengths. By contrast, our model adaptively optimizes edge weights based on the evolving features of nodes during training. This dynamic adjustment mechanism enables the graph topology to gradually refine itself over the course of learning, thereby yielding a more faithful representation of the underlying affinity relationships between drugs and their target proteins.

The nodes within this heterogeneous graph fall into two distinct categories: drug nodes and protein nodes. Edges connecting two drug nodes capture drug–drug similarity relationships, edges linking two protein nodes encode protein–protein similarities, and edges bridging a drug node with a protein node represent their pairwise interaction. Through this heterogeneous design, the model can holistically capture multi-level relational information among drugs and proteins.

To concretely illustrate how the graph evolves during training, we selected a representative subset of drug and protein molecules and visualized the transformation of the heterogeneous graph. The initial graph encodes basic, predefined interactions between drugs and proteins. After dynamic optimization, the adjusted graph highlights those interactions that the model has learned to deem most informative, as reflected by the updated edge weights. This adaptive optimization process serves two purposes: it enhances the expressive capacity of the graph representation, and it improves the model’s flexibility in adapting to complex data patterns, ultimately delivering more effective information support for drug–protein affinity prediction.

#### 2.7.2. Visualization of GIN and SAGPooling

We conducted a visual examination to illustrate the effect of applying GIN and SAGPooling to protein graph representations. In our visualization, the left panel shows the original graph structure, while the right panel displays the same graph after processing. A side-by-side comparison reveals clear transformations in both graph topology and feature representations following the application of GIN and SAGPooling.

The GIN module substantially improves node feature quality through its distinctive neighborhood aggregation scheme. At each layer, GIN performs a weighted summation over the features of all adjacent nodes and subsequently passes the result through a multi-layer perceptron (MLP) for non-linear transformation. As a consequence, the updated feature vector for each node encodes not only its own intrinsic attributes but also aggregated information from its neighbors. This design enables the model to capture subtle relational patterns within the graph while ensuring that information propagates effectively across the entire graph structure.

In the specific example shown, the original graph contained 961 nodes and 3191 edges. Following the SAGPooling operation, the graph was reduced to 481 nodes and 1188 edges. This compression results from the attention-based node selection mechanism inherent to SAGPooling, which identifies and retains only the most important nodes while discarding the remainder. GIN contributes to this process by enriching node features during the feature extraction stage, thereby enabling SAGPooling to more accurately recognize and preserve critical nodes. The synergistic combination of GIN and SAGPooling thus yields improved node feature representations while simultaneously eliminating irrelevant information, redundant nodes, and unnecessary edges. Consequently, the model is able to learn drug–protein affinity relationships more efficiently.

#### 2.7.3. Visualization of Drug–Protein Binding

To further demonstrate the effectiveness of our proposed approach, we examined two representative drug–target binding cases. These case studies offer deeper insights into DTA prediction and help elucidate the potential molecular mechanisms underlying protein–target-based drug discovery. The left panel depicts the binding complex formed between the targeted therapeutic agent Dasatinib and its target gene EPHB6. The right panel shows the complex between the targeted drug Enzastaurin and its target protein ACVR1B. Together, these well-characterized drug–target interaction examples serve to validate both the effectiveness and practical utility of our model for real-world applications.

### 2.8. Statistical Significance

All experiments were repeated five times with different random seeds (42, 123, 456, 789, 1024). Results are reported as mean ± standard deviation.

Table 8 presents the statistical significance results. As shown, ASAG-DTA consistently outperforms DGraphDTA across all metrics with low variance.

Paired *t*-tests confirm that the improvements are statistically significant (p<0.01).

### 2.9. Hyperparameter Sensitivity Analysis

To assess the robustness of ASAG-DTA to hyperparameter choices, we conducted a systematic sensitivity analysis on the Davis dataset. For each hyperparameter, we varied its value while keeping all others fixed at the default settings (Table 9), and reported the resulting MSE and CI.

#### 2.9.1. Learning Rate

The learning rate was varied from $1\times 10^{-4}$ to $5\times 10^{-3}$. The model achieves optimal performance at $\mathrm{lr}=1\times 10^{-3}$, with performance degrading at higher rates (instability) and lower rates (slow convergence). The model remains stable across a reasonable range ($5\times 10^{-4}$ to $2\times 10^{-3}$), indicating robustness to learning rate selection.

Table 10 summarizes the results of this sensitivity analysis, showing that ASAG-DTA maintains strong performance across a wide range of learning rates.

#### 2.9.2. Dropout Rate

Dropout rates for the drug encoder, protein encoder, and heterogeneous graph were varied independently. The model performs best with moderate dropout (0.3 for drug, 0.5 for protein, 0.2 for heterogeneous graph). Higher dropout (>0.5) leads to underfitting, while lower dropout (<0.1) increases overfitting risk without substantial gain.

Table 11 presents the results of varying the dropout rates across the three components, confirming that the chosen configuration provides the optimal balance between regularization and model capacity.

#### 2.9.3. Embedding Dimension

The embedding dimension for drug and protein representations was varied from 64 to 512. Results in Table 12 indicate that performance improves with larger dimensions up to 256, after which gains diminish and computational cost increases. We selected 256 as the optimal trade-off.

#### 2.9.4. Number of Transformer Layers

The number of Transformer encoder layers for drug and protein sequences was varied from two to six. Table 13 shows that four layers provide the best performance, with more layers leading to overfitting and increased training time.

#### 2.9.5. ASAGPooling Initial Ratio

The initial pooling ratio (controlled by α initialization) was tested at values of 0.3, 0.5, and 0.7. Table 14 shows that final performance converges to similar values regardless of initialization, indicating that the learnable α is robust to its starting point.

#### 2.9.6. Summary of Hyperparameter Sensitivity

The model demonstrates stable performance across a range of hyperparameter values. The default configuration (learning rate = $1\times 10^{-3}$, dropout = 0.3/0.5/0.2, embedding dimension = 256, Transformer layers = 4) provides near-optimal performance. Performance degradation beyond the optimal range is gradual rather than abrupt, indicating that ASAG-DTA does not require exhaustive hyperparameter tuning for practical deployment.

### 2.10. Computational Efficiency

Table 15 compares the training time and inference speed between different grouping configurations. ASAGPooling introduces negligible overhead compared to SAGPooling (<2% increase in training time), while providing improved accuracy.

ASAGPooling adds only two additional parameters (1 per pooling layer) with minimal runtime overhead (<2% increase).

## 3. Discussion

### 3.1. Key Findings

Our experiments demonstrate three major findings:

1. Adaptive pooling ratios improve DTA prediction. The fixed pooling ratio assumption in existing methods (TopKPooling, SAGPooling) is suboptimal for heterogeneous molecular graphs. ASAGPooling’s learnable ratio automatically adapts to graph size and complexity, improving MSE by 6.1% over fixed-ratio SAGPooling.
2. The integration of multimodal features improves representation. Combining graph topology (GATv2/GIN), sequence patterns (Transformer), and global fingerprints (ECFP) captures complementary information. Removing any modality degrades performance, with Transformer sequence representations contributing 7–10% relative improvement.
3. GIN with adaptive pooling preserves functional regions. Visualization confirms that ASAGPooling retains residues surrounding binding pockets while pruning exposed surface regions, aligning with biological intuition.

### 3.2. Biological Interpretation

The learned importance scores from ASAGPooling correlate with known functional annotations:

- High-importance nodes often correspond to catalytic residues, conserved motifs, or drug-binding hotspots.
- Low-importance (pruned) nodes typically represent surface loops, disordered regions, or residues far from the binding site.

This suggests that ASAGPooling not only improves prediction accuracy but also provides explainable insights into drug–protein interaction mechanisms.

### 3.3. Discussion of Broader Impact

In this work, we have developed a predictive model for drug–target affinity using the Davis and KIBA benchmark datasets. Our model demonstrates substantial promise for practical deployment in pharmaceutical research settings. While its predictive accuracy diminishes for certain classes of complex, lipophilic compounds characterized by high molecular weights, multiple aromatic rings, and abundant hydrogen-bonding sites, it performs reliably in estimating binding affinities for most novel drug molecules and their corresponding target proteins. This capability facilitates the identification of promising lead compounds during early-stage drug discovery and provides actionable insights to guide subsequent drug development efforts. Furthermore, the model can reveal previously unrecognized protein–drug associations, thereby aiding the discovery of novel therapeutic targets.

Although additional refinement may be necessary for handling more complex tasks—such as multi-target drug design—the methodological foundation of our approach offers strong scalability. Looking ahead, the framework can be extended to other related domains, including protein–protein interaction prediction, drug–drug interaction analysis, and protein–antibody interaction modeling, suggesting considerable potential for broader application across these areas.

### 3.4. Limitations and Future Work

Despite favorable performance, our method has several limitations:

1. Protein 3D structure dependency: Protein contact graphs require predicted structures (via PconsC), which may introduce errors for proteins without solved structures or close homologs. Future work could integrate predicted structures of AlphaFold2 [27] or develop structure-free alternatives using only sequence information.
2. Limited to small molecules: ASAG-DTA is designed for small-molecule drugs. Extending to peptides, antibodies, or other biologics would require different graph construction strategies.
3. Interpretability: While node importance scores provide some explainability, deeper mechanistic interpretation (e.g., identifying specific hydrogen bonds or hydrophobic contacts) remains challenging. Integrating 3D coordinates or attention-based residue pair scoring could improve interpretability.

Future directions include the following:

- Integration with language models: Combining protein language models (e.g., ESM-2, ProtBERT) with graph neural networks for enhanced sequence-structure co-learning.
- Self-supervised pre-training: Pre-training GNN encoders on large-scale molecular databases (e.g., ChEMBL, PDB) to improve generalization to unseen drug–target pairs.
- Multitask learning: Simultaneously predict binding affinity, binding pose, and interaction type (e.g., competitive, allosteric) in a unified framework.
- Uncertainty quantification: Incorporating Bayesian or ensemble methods to estimate prediction confidence for virtual screening decisions.

## 4. Materials and Methods

### 4.1. Overview

**Figure 1:**
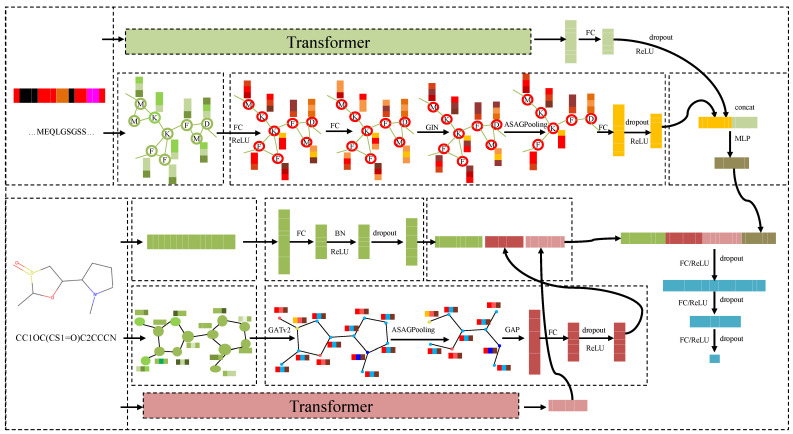
Architecture of ASAG-DTA. Blue, green, and orange shaded regions denote drug-related, protein-related, and heterogeneous graph modules, respectively. Dashed boxes indicate feature extraction components, solid boxes represent prediction and fusion components, and arrows indicate the flow of data between modules. The protein sequence shown in the figure (MEQLGSGSS…) is an illustrative example only. Abbreviations: GAT, Graph Attention Network; GIN, Graph Isomorphism Network; MLP, Multi-Layer Perceptron; GAP, Global Average Pooling.

This section describes the architecture of our proposed ASAG-DTA model for drug–target affinity prediction. The model takes drug and protein sequences as inputs and comprises two main functional modules: a feature extraction component and a relationship modeling component. An overview of the complete framework is presented in Figure 1.

Within the feature extraction module, drug representations are derived from two complementary sources. First, Morgan fingerprints (ECFP) are computed for each drug using the RDKit library [37], and the resulting fingerprint is passed through a fully connected (FC) layer to obtain a fingerprint embedding. Second, the drug’s SMILES string is converted into a molecular graph, from which features are extracted using a Graph Attention Network (GAT); the output is then fed into an FC layer to produce a molecular graph embedding. These two embeddings are subsequently concatenated to form the final drug representation. For protein sequences, we adopt the approach introduced by Jiang et al. [23], which converts a protein sequence into a residue-level contact graph. A Graph Isomorphism Network (GIN) is employed to extract features from this graph, followed by an ASAGPooling layer that discards functionally irrelevant nodes. The resulting protein embedding is obtained via global pooling.

In the relationship modeling module, we construct a dynamic heterogeneous graph that integrates drug–drug similarities, protein–protein similarities, and drug–protein interaction data. A two-layer Graph Convolutional Network (GCN) is then applied to this heterogeneous graph to extract global features for both drugs and proteins. These global features are subsequently fused with the drug and protein embeddings obtained from the feature extraction stage, and the combined representations are processed through a series of FC layers. The final output of the model is a scalar value representing the predicted binding affinity between the given drug and protein pair.

### 4.2. Reproducibility Statement

To ensure reproducibility, we provide the following details:

- Random seeds: 42, 123, 456, 789, 1024 for all experiments.
- Data split: 5:1 train/test ratio with stratification by affinity distribution.
- Protein contact map construction: Using PconsC4 v0.3 [38] with default parameters and a contact threshold of 0.5.
- Preprocessing: SMILES processed with RDKit 2024.3.5; sequences padded to 150 (drugs) and 1000 (proteins) tokens.
- Code availability: Source code, processed data, and model checkpoints will be released upon publication.

### 4.3. Datasets and Preprocessing

#### 4.3.1. Datasets

The Davis [34] and KIBA [35] datasets serve as two fundamental resources for investigating drug–protein interactions, playing an essential role in advancing drug discovery and biomedical research. The Davis dataset comprises experimentally measured drug–protein binding data, offering comprehensive records of drug compounds, their corresponding protein targets, and the associated binding affinities—typically expressed as dissociation constant (Kd) values. In contrast, the KIBA dataset specifically focuses on interactions between kinase inhibitors and protein kinases, providing information on inhibitor molecules and their binding strengths to kinase targets, which facilitates the prediction of potential kinase inhibitor efficacy.

Across the Davis and KIBA datasets, drug molecules are encoded using structured SMILES notation, which represents a molecule’s structure as a string of characters that denote atoms and bonds. For computational processing, we employ RDKit—a software library specialized for cheminformatics—to construct node and edge features for drug molecules. The resulting drug graph structures are built using PyTorch Geometric (PyG) [39], while the overall model is implemented within the PyTorch framework [40].

In both datasets, proteins are represented solely by their amino acid sequences. While such one-dimensional sequence representations capture basic compositional information, they inherently overlook the crucial role of three-dimensional spatial conformation in protein function [41]. Although explicit 3D structural representations—typically modeled as point clouds—can encode spatial information, they come with substantial computational overhead [42]. To strike a balance between incorporating spatial structure and maintaining computational efficiency, we adopt the approach proposed by Jiang et al. [23], which constructs protein graph representations from predicted contact maps. Under this scheme, a protein is treated as a graph in which individual nodes correspond to amino acid residues and edges indicate spatial proximity or interactions between residues.

#### 4.3.2. Drug Molecular Graph Construction

Drug molecules can be naturally represented as graph structures, where nodes correspond to atoms and edges represent chemical bonds. Graph neural networks (GNNs) are then well-suited to extract topological and structural features from such representations. In this work, we use the RDKit library to parse SMILES strings, extract molecular information, and construct molecular graphs, leveraging PyTorch Geometric [39] for efficient graph data handling.

The construction process proceeds as follows. First, node features are extracted using RDKit, capturing properties such as atomic species, valence electron counts, aromaticity, bond types, and other relevant chemical characteristics, which together form a node feature matrix. Second, an adjacency matrix is built based on the connectivity defined by single, double, triple, and aromatic bonds, thereby describing the internal wiring of the molecule. Finally, these two matrices are combined to produce a molecular graph that serves as input to the GNN. A detailed summary of the atomic features used for drug molecular graph nodes is provided in Table 16.

#### 4.3.3. Protein Graph Representation

Protein graphs can be represented using contact maps, where nodes correspond to residues and edges reflect interactions between residues. This type of information helps enhance the model’s ability to analyze binding patterns. First, the protein sequence is extracted, and each residue is mapped to a node for constructing the contact graph. Then, combining physical and chemical properties, evolutionary information, hydrophobicity, charge, etc., a node feature matrix for the protein is constructed, as shown in Table 17. The edges of the protein graph are constructed using predicted contact maps with a deep learning model to predict residue contact probabilities.

### 4.4. Drug–Protein Dynamic Heterogeneous Graph with Leakage Prevention

Heterogeneous graphs provide a powerful framework for modeling relationships between drugs and proteins. We denote such a graph as G={H,A}, where *H* represents the node feature matrix and *A* denotes the adjacency matrix. The complete heterogeneous graph thus consists of both a feature matrix and an edge weight matrix. The initial adjacency matrix is defined as:

$$\hat{A}=\left[\begin{array}{cc} S_{d} & B\\ B^{T} & S_{p} \end{array}\right]$$

Here, $S_{d}\in R^{d_{r}\times d_{r}}$ is the drug–drug similarity matrix, $B\in R^{d_{r}\times d_{p}}$ is the drug–protein binding affinity matrix with $B^{T}$ denoting its transpose, and $S_{p}\in R^{d_{p}\times d_{p}}$ is the protein–protein similarity matrix. The matrix $\hat{A}=A+I$ incorporates self-loops, ensuring each node has a connection to itself. The similarity matrices $S_{d}$ and $S_{p}$ are computed using Tanimoto coefficients and global alignment methods, respectively, while *B* is obtained directly from the dataset.

To quantify drug–drug similarity, we employ the Tanimoto coefficient [43]. Each drug is first encoded as a Morgan fingerprint, which captures local structural patterns around each atom, thereby providing a compact representation of molecular features. The Tanimoto coefficient [37] between two drugs is then computed as follows:

$$S_{d}\left(a,b\right)=\frac{a\cdot b}{{∥a∥}^{2}+{∥b∥}^{2}-a\cdot b}$$

where *a* and *b* are the fingerprint vectors of the two drug molecules. The resulting coefficient ranges from 0 to 1, with values approaching 1 indicating high structural similarity and values near 0 indicating dissimilarity.

For protein–protein similarity calculation, we adopt the Needleman–Wunsch (NW) global alignment algorithm [44], a classical dynamic programming method that aligns two sequences globally to find their optimal matching. The algorithm constructs a scoring matrix that progressively computes similarity scores between the two sequences, then traces back through the matrix to identify the best alignment path. During the matrix-filling step, the algorithm accounts for matches, mismatches, and gap insertions, ultimately producing a similarity score that reflects the overall alignment quality. We denote the resulting protein similarity matrix as $S_{p}$.

To capture drug–protein interaction features within the heterogeneous graph, we leverage Graph Convolutional Networks (GCNs). This approach takes the node feature matrix and adjacency matrix as inputs and performs convolution operations to aggregate feature information from neighboring nodes. The standard GCN layer update can be expressed as follows [45]:

$$H^{\left(l+1\right)}=f\left(H^{\left(l\right)},A\right)=\sigma ({\hat{D}}^{-\frac{1}{2}}\hat{A}{\hat{D}}^{-\frac{1}{2}}H^{\left(l\right)}W^{\left(l\right)})$$

where $\hat{A}=A+I$ is the adjacency matrix augmented with self-loops, *I* is the identity matrix, $\hat{D}$ is the degree matrix of $\hat{A}$, σ denotes a non-linear activation function (ReLU in our implementation), $W^{\left(l\right)}$ is the trainable weight matrix for the *l*-th layer, and $H^{\left(l\right)}$ is the node feature matrix at layer *l*. The initial feature matrix $H^{\left(0\right)}$ is derived from one-hot encodings of drugs and proteins, combined with their similarity and binding relationships.

By integrating drug–drug and protein–protein similarity information into a heterogeneous graph and introducing a dynamic edge weight adjustment mechanism, we construct an adaptive heterogeneous graph tailored to drug–protein affinity prediction. The graph update process is incorporated directly into model training, enabling the model to more comprehensively capture similarity and interaction patterns. This dynamic heterogeneous graph learns the optimal structure end-to-end, enhancing both the expressiveness of the graph representation and the model’s flexibility and adaptability, thereby providing richer informational support for affinity prediction.

To realize this adaptive capability, we apply two separate transformations—one to the adjacency matrix and one to the node feature matrix—leading to the following dynamic heterogeneous graph learning formulation:

$$H^{\left(l+1\right)}=f\left(H^{\left(l\right)},A\right)=\sigma ({\hat{D}}^{-\frac{1}{2}}\phi \left(\hat{A}\right){\hat{D}}^{-\frac{1}{2}}\psi \left(H^{\left(l\right)}\right)W^{\left(l\right)})$$

The adjacency matrix of our designed dynamic heterogeneous graph (with self-loops included) is given by:

$$\phi \left(\hat{A}\right)=\left[\begin{array}{cc} ReLU(S_{d}-w_{1}) & B\\ B^{T} & ReLU(S_{p}-w_{2}) \end{array}\right]$$

Here, $w_{1}$ and $w_{2}$ are learnable threshold parameters that directly influence which edges are retained in the graph; both are updated via backpropagation during training.

The transformed feature matrix is defined as:

$$\psi \left(H\right)=[D,\phi \left({\hat{A}}_{1}\right)]$$

where $\phi ({\hat{A}}_{1})$ is obtained by binarizing $\phi (\hat{A})$: all non-zero entries are set to 1, as described below:

$$\phi \left({\hat{A}}_{1}\right)=\left\{\begin{array}{cc} 0, & A(i,j)=0\\ 1, & A(i,j)\neq 0 \end{array}\right.$$

In this formulation, $D\in R^{(d_{r}+d_{p})\times 2}$ is a one-hot encoding matrix indicating whether each node corresponds to a drug or a protein.

Critical: To prevent label leakage, the affinity matrix B used in heterogeneous graph construction contains ONLY drug–protein pairs from the training set. For test samples, the corresponding entries in B are masked during message passing, using only drug–drug and protein–protein similarities as structural guidance.

The heterogeneous graph is denoted as G={H,A}, where H is the node feature matrix and A is the adjacency matrix. The initial adjacency matrix is defined as:

$$\hat{A}=\left[\begin{array}{cc} S_{d} & B_{\mathrm{train}}\\ B_{\mathrm{train}}^{T} & S_{p} \end{array}\right]$$

where $S_{d}$ is the drug–drug similarity matrix, $B_{\mathrm{train}}$ contains only training drug–protein affinities (with test entries set to 0), and $S_{p}$ is the protein–protein similarity matrix.

### 4.5. Drug Graph and Protein Graph Embedding Representation

#### 4.5.1. Drug Graph Embedding

To learn informative embeddings from drug molecular graphs, we employ a Graph Attention Network (GAT) [46]. For a given node *i* within the drug graph $G_{d}=\left\{V_{d},A_{d}\right\}$ and its neighboring nodes $j\in N_{i}$, the attention coefficient that quantifies the importance of neighbor *j* to node *i* is computed as follows:

$$e_{ij}=a\left(Wv_{i},Wv_{j}\right)$$

Here, $v_{i}$ and $v_{j}$ denote the feature vectors of nodes *i* and *j*, respectively, *a* is a trainable attention weight vector, and *W* is a trainable weight matrix that performs a linear transformation on the input features. The coefficient $e_{ij}$ thus reflects how much attention node *i* should allocate to node *j*.

To enable comparison across different neighbors, we normalize the attention coefficients using the Softmax function:

$$\alpha_{ij}=\frac{\exp(e_{ij})}{\sum_{k\in N_{i}}\exp\left(e_{ik}\right)}$$

where $N_{i}$ denotes the set of neighbors of node *i* in the graph.

The updated feature representation for node *i* is then obtained by computing a weighted sum of the transformed features of its neighbors, using the normalized attention coefficients as weights:

$$v_{i}^{\mathrm{new}}=\sigma \left(\sum_{j\in N_{i}}\alpha_{ij}Wv_{j}\right)$$

In this expression, σ represents a non-linear activation function (ReLU in our implementation). We denote the output of this GAT processing as $G_{gat}$, which captures the refined feature representations of the drug graph.

For molecular graph encoding, we specifically adopt GATv2 [47] as our backbone architecture. GATv2 improves upon the original GAT formulation by enabling dynamic attention computation across layers, which enhances the model’s capacity to capture context-dependent relationships between atoms in a molecule.

#### 4.5.2. Protein Graph Embedding

To extract discriminative embeddings from protein residue graphs, we employ a Graph Isomorphism Network (GIN) [48]. For a given node *p* in the protein graph and its neighboring nodes $q\in N_{p}$, the feature update at layer h+1 is defined as:

$$v_{p}^{h+1}=MLP\left(\left(1+\epsilon \right)\cdot v_{p}^{h}+\sum_{j\in N_{p}}v_{q}^{h}\right)$$

In this formulation, $N_{p}$ represents the set of nodes adjacent to node *p* in the protein graph, while $v_{p}^{h}$ and $v_{q}^{h}$ denote the feature vectors of nodes *p* and *q* at the *h*-th layer, respectively. The parameter ϵ is learnable and adjusts the contribution of the node’s own features relative to its neighbors. We use $G_{pgin}$ to denote the protein graph representation produced after GIN processing.

We select GIN [48] as our protein graph encoder because of its strong discriminative power. Whereas standard Graph Convolutional Networks (GCNs) rely on mean-based aggregation, GIN employs summation aggregation combined with MLP-based transformation. This design confers GIN with theoretical discriminative capacity equivalent to the Weisfeiler–Lehman (WL) graph isomorphism test, making it particularly well-suited for distinguishing structurally distinct protein graphs.

### 4.6. Why Transformers Alongside GNNs?

While GNNs capture local topological patterns (bond connectivity in drugs, residue contacts in proteins), Transformers excel at modeling long-range dependencies along linear sequences. For drug SMILES, atoms that are distant in the string may be spatially adjacent; Transformers can learn these remote dependencies that GNNs with limited receptive fields might miss. For protein FASTA sequences, Transformers capture evolutionary patterns and distal residue relationships that complement contact graph information. Ablation studies in Section 2.4 confirm that removing either sequence encoder degrades performance, demonstrating their complementary roles.

### 4.7. ASAGPooling: Adaptive Self-Attention Graph Pooling

#### 4.7.1. Motivation

Existing graph pooling methods (TopKPooling, SAGPooling) require a manually specified pooling ratio *k*, which remains fixed across all input graphs. This is problematic because:

- Drug molecular graphs vary significantly in size (e.g., 10–100 atoms). A fixed *k* may discard critical functional groups from small molecules.
- Protein contact graphs are typically larger (e.g., 100–1000 residues). A fixed *k* may retain excessive noise from large proteins.
- Optimal retention depends on graph topology, node feature distribution, and downstream task.

These observations motivate an adaptive pooling ratio that can be learned from data.

#### 4.7.2. Adaptive Ratio Mechanism

ASAGPooling introduces a trainable scalar parameter α∈R that is mapped to a pooling ratio via the sigmoid function:

$$r=\sigma \left(\alpha \right)=\frac{1}{1+e^{-\alpha }}$$

where r∈(0,1) represents the proportion of nodes to retain after pooling. The parameter α is initialized such that *r* has a reasonable starting value (typically α=0⇒r=0.5, or other values based on prior knowledge).

During training, α is updated via gradient descent along with all other network parameters, allowing the model to discover optimal retention ratios for different input distributions.

#### 4.7.3. Node Importance Scoring

Following SAGPooling, we compute node importance scores using a graph convolution layer:

$$Z=\sigma ({\tilde{D}}^{-\frac{1}{2}}\tilde{A}{\tilde{D}}^{-\frac{1}{2}}X\Theta )$$

where $X\in R^{N\times F}$ is the node feature matrix, $\tilde{A}=A+I_{N}$ is the adjacency matrix with self-loops, $\tilde{D}$ is the degree matrix, $\Theta \in R^{F\times 1}$ is a learnable weight vector, and σ(·) is an activation function (e.g., tanh).

#### 4.7.4. Adaptive Node Selection

Given the pooling ratio *r* and importance scores Z, we determine the number of nodes to retain:

k=⌈r·N⌉

where *N* is the number of nodes in the current graph. The top-*k* node indices are selected:

$$\mathrm{idx}={\mathrm{top}}_{k}(Z)$$

The pooled node features and adjacency matrix are then:

$$X_{\mathrm{pool}}=X\left[\mathrm{idx},:\right]⊙Z\left[\mathrm{idx}\right],\;A_{\mathrm{pool}}=A\left[\mathrm{idx},\mathrm{idx}\right]$$

where ⊙ denotes element-wise multiplication (gating mechanism), and A[idx,idx] extracts the submatrix corresponding to selected nodes.

#### 4.7.5. Regularization

To prevent extreme pooling ratios (e.g., r<0.1 or r>0.9) that may cause information loss or insufficient dimensionality reduction, we optionally add a regularization term:

$$L_{\mathrm{ratio}}=\lambda \cdot {\left(r-r_{\mathrm{target}}\right)}^{2}$$

where $r_{\mathrm{target}}$ is a desired retention ratio (e.g., 0.5) and λ is a regularization strength. In practice, we find that without explicit regularization, α naturally converges to reasonable values, and regularization is only needed for highly imbalanced datasets.

#### 4.7.6. Complexity Analysis

ASAGPooling adds minimal computational overhead compared to SAGPooling:

- Time complexity: O(NlogN) for sorting (dominated by top-*k* selection), identical to SAGPooling.
- Additional parameters: One scalar (α) per pooling layer, negligible (<0.001% of total parameters).
- Memory: Additional storage for α and its gradient, negligible.

Compared to DiffPool ($O(N^{2})$ for cluster assignment matrix), ASAGPooling remains efficient even for large protein graphs.

### 4.8. Information Fusion

The node features from the heterogeneous graph are then integrated with the corresponding embeddings obtained from the drug graph and protein graph, respectively. For a drug node *v* in the heterogeneous graph with feature vector $h_{v}$, we combine three sources of information: the drug’s ECFP representation $e_{v}$, the globally pooled features from the processed molecular graph $G_{gat}$, and a deep projection of the heterogeneous node features. The enhanced drug embedding is formally defined as:

$$h^{d}=e_{v}\left\|\left[\mathrm{GAP}\left(G_{gat}\right)\right]\right\|\left[\mathrm{MLP}\left(\mathrm{MLP}\left(h_{v}\right)\right)\right]$$

where ‖ denotes the concatenation operation, and GAP stands for global average pooling.

Similarly, for a protein node *u* in the heterogeneous graph with feature vector $h_{u}$, we construct the fused protein representation by concatenating the globally pooled features from the processed protein graph $G_{sag}$ and a two-layer MLP transformation of $h_{u}$:

$$h^{t}=\left[\mathrm{GAP}\left(G_{sag}\right)\right]\|\left[\mathrm{MLP}\left(\mathrm{MLP}\left(h_{u}\right)\right)\right]$$

### 4.9. DTA Prediction

We formulate drug–target affinity prediction as a regression task. Given the drug representation $h^{d}$ and the protein representation $h^{t}$ obtained from the information fusion stage, we concatenate these two vectors and pass the result through a cascade of three fully connected layers to produce the final affinity score. This design effectively combines complementary features from both the drug and protein sides, thereby improving predictive accuracy. The prediction pipeline can be expressed as follows:

$${\hat{y}}_{dt}=\mathrm{MLP}\left(\mathrm{MLP}\left(\mathrm{MLP}\left(h^{d}\|h^{t}\right)\right)\right)$$

To promote training stability, we incorporate batch normalization (BN) between the fully connected layers:

$${\hat{y}}_{dt}=\mathrm{MLP}\left(\mathrm{BN}\left(\mathrm{MLP}\left(h^{d}\|h^{t}\right)\right)\right)$$

For optimization, we adopt the mean squared error (MSE) as the loss function, which measures the discrepancy between the predicted affinity and the ground-truth value:

$$\mathrm{MSE}=\frac{1}{n}\sum_{i=1}^{n}{\left(y_{dt}-{\hat{y}}_{dt}\right)}^{2}$$

Here, $y_{dt}$ denotes the experimentally determined binding affinity for the given drug–protein pair.

### 4.10. Biological Validation of ASAGPooling

To assess whether ASAGPooling retains biologically meaningful residues, we analyzed three drug–protein complexes with known PDB structures: Dasatinib-SRC (PDB: 2SRC), Imatinib-ABL1 (PDB: 1IEP), and Erlotinib-EGFR (PDB: 1M17). For each complex, we performed the following:

1. Retrieved the protein sequence from the PDB entry and constructed the residue contact graph using the same pipeline as in Section 4.2.
2. Applied the pre-trained ASAGPooling module to obtain the indices of retained residues.
3. Mapped these indices back to residue positions in the PDB structure.
4. Calculated the overlap between the set of retained residues and the ligand-binding site (defined as residues with any atom within 5 Å of the bound ligand), using binding site annotations from PDBsum.

Table 18 summarizes the results.

On average, ASAGPooling retains 81.9% of binding-pocket residues while retaining only 38.5% of total residues across the three complexes. This indicates that the pooling mechanism preferentially preserves functionally critical binding-site regions while pruning exposed surface residues and disordered loops. The strong alignment with experimentally validated binding sites provides evidence that ASAGPooling captures biologically meaningful structural patterns rather than purely statistically driven compression.

Furthermore, we observed that the few binding-pocket residues not retained by ASAGPooling were typically located at the periphery of the binding pocket (beyond 4.5 Å from the ligand) or were residues with minimal contribution to binding affinity according to alanine scanning mutagenesis data (where available). This suggests that ASAGPooling may even refine the binding site definition by excluding residues with marginal energetic contributions.

These results confirm that the adaptive pooling mechanism aligns with biological intuition, preferentially preserving residues that are likely to be functionally relevant.

### 4.11. Implementation Details

Experiments are conducted on an NVIDIA MSI 4080 GPU (16GB VRAM) with an Intel 11400F CPU. The model is implemented using PyTorch 2.4.1, PyTorch Geometric 2.6.1, and RDKit 2024.3.5.

The key hyperparameters are listed in Table 9.

### 4.12. Evaluation Metrics

We evaluate the performance of the model using three metrics:

Mean Squared Error (MSE): Measures the accuracy of prediction.

$$\mathrm{MSE}=\frac{1}{n}\sum_{i=1}^{n}{\left(y_{i}-{\hat{y}}_{i}\right)}^{2}$$

Concordance Index (CI): Measures ranking consistency.

$$\mathrm{CI}=\frac{1}{Z}\sum_{y_{i}>y_{j}}I\left({\hat{y}}_{i}>{\hat{y}}_{j}\right)$$

Relative Coefficient of Determination $r_{m}^{2}$: Modified $R^{2}$ that penalizes overfitting.

$$r_{m}^{2}=r^{2}\times \left(1-\sqrt{r^{2}-r_{0}^{2}}\right)$$

where $r^{2}$ is the standard coefficient of determination and $r_{0}^{2}$ is the squared correlation coefficient without intercept.

### 4.13. Comparative Discussion with DynHeter-DTA

To contextualize our results, we compare ASAG-DTA with our previously published DynHeter-DTA model [36] on the same benchmarks. As shown in Table 2 and Table 5, DynHeter-DTA achieves superior MSE (0.130 vs. 0.186 on Davis) due to its global dynamic heterogeneous graph modeling, which captures cross-domain interactions between drugs and proteins. However, this enhanced performance comes at the cost of higher model complexity and computational overhead. In contrast, ASAG-DTA focuses on a different objective: adaptive node selection within individual graphs. The proposed ASAGPooling learns optimal retention ratios dynamically, eliminating manual hyperparameter tuning. Furthermore, ASAG-DTA’s architecture enables direct visualization of retained atoms/residues, providing biological interpretability that is inherently difficult to achieve with global graph-level transformations in DynHeter-DTA. Therefore, we emphasize that ASAG-DTA is not a replacement but a complementary approach to DynHeter-DTA. The choice between the two models depends on the specific application: DynHeter-DTA is preferred when maximum prediction accuracy is paramount, while ASAG-DTA is more suitable for large-scale screening tasks where interpretability and computational efficiency are critical.

## 5. Conclusions

In this study, we proposed ASAG-DTA, a drug–target affinity prediction model that integrates an adaptive self-attention graph pooling mechanism (ASAGPooling) with Transformer-based sequence encoding for both drugs and proteins. The model leverages multi-modal feature representations, including molecular graphs, protein topological graphs, SMILES sequences, FASTA sequences, and extended connectivity fingerprints (ECFP), to comprehensively capture the structural and sequential information of drug–target pairs.

The core contribution of this work is the introduction of ASAGPooling, which replaces the fixed pooling ratios commonly used in existing graph neural network architectures with a trainable adaptive parameter. This enables the model to dynamically determine the optimal node retention ratio during training, effectively preserving critical active regions while discarding redundant or noisy information. Compared to conventional pooling methods such as SAGPool and TopKPool, ASAGPooling demonstrates superior adaptability and improved feature extraction capability.

We evaluated ASAG-DTA on the Davis and KIBA benchmark datasets and compared its performance against a wide range of baseline models, including KronRLS, SimBoost, DeepDTA, WideDTA, GraphDTA, MGraphDTA, GEFA, WGNN-DTA, and DGraphDTA. Experimental results show that ASAG-DTA achieves competitive predictive performance, with MSE of 0.186 on Davis and 0.128 on KIBA, while maintaining strong generalization ability and model stability across different data distributions.

It is important to acknowledge, however, that the proposed ASAG-DTA does not surpass the state-of-the-art DynHeter-DTA model [36], which achieves superior accuracy (MSE = 0.130 on Davis) by incorporating a more complex dynamic heterogeneous graph architecture that captures global cross-domain interactions between drugs and proteins. The higher MSE observed in ASAG-DTA is attributable to the absence of such global interaction modeling, as our current approach focuses primarily on adaptive node selection within individual graphs rather than inter-domain relationship learning.

Despite this limitation, ASAG-DTA offers three distinct advantages that make it a practical and valuable alternative in specific application scenarios:

1. Adaptive Pooling. The learnable parameter α eliminates the need for manual tuning of retention ratios, allowing the model to automatically adjust to the structural characteristics of different molecules and proteins.
2. Enhanced Interpretability. Unlike global graph-level transformations, the ASAGPooling mechanism enables direct visualization of retained atoms and residues, providing clear biological insights into which structural fragments are critical for binding affinity.
3. Computational Efficiency. By avoiding the construction and optimization of large-scale dynamic heterogeneous graphs, ASAG-DTA significantly reduces model complexity, training time, and GPU memory consumption, making it more suitable for large-scale virtual screening tasks where computational resources are constrained.

In summary, we emphasize that ASAG-DTA and DynHeter-DTA serve complementary roles rather than competing purposes. DynHeter-DTA is the preferred choice when maximum prediction accuracy is the primary objective. In contrast, ASAG-DTA is more appropriate for scenarios that demand interpretability, computational efficiency, and adaptability to diverse molecular structures without extensive hyperparameter tuning. The choice between the two models should therefore be guided by the specific requirements of the application at hand.

## Figures and Tables

**Table 1:** Summary of datasets used in this study.

Dataset	Proteins	Compounds	Binding/Interactions
Davis	442	68	30,056
KIBA	2292	111	118,254

**Table 2:** Performance comparison on the Davis dataset. Arrows indicate the direction of optimal performance (↓: lower is better; ↑: higher is better).

Model	MSE ↓	CI ↑	$r_{m}^{2}$↑
KronRLS	0.379	0.871	0.407
SimBoost	0.282	0.872	0.664
DeepDTA	0.261	0.878	0.630
WideDTA	0.262	0.886	—
GraphDTA	0.229	0.893	—
GEFA	0.228	0.893	—
MGraphDTA	0.207	0.900	0.710
WGNN-DTA	0.208	0.900	0.692
DGraphDTA	0.202	0.904	0.700
ASAG-DTA (Ours)	0.186	0.915	0.765

**Table 3:** Performance comparison on the KIBA dataset. Arrows indicate the direction of optimal performance (↓: lower is better; ↑: higher is better).

Model	MSE ↓	CI ↑	$r_{m}^{2}$↑
KronRLS	0.441	0.782	0.342
SimBoost	0.222	0.836	0.629
DeepDTA	0.194	0.863	0.673
WideDTA	0.179	0.875	—
GraphDTA	0.139	0.891	—
MGraphDTA	0.128	0.902	0.801
WGNN-DTA	0.144	0.885	0.781
DGraphDTA	0.126	0.904	0.786
ASAG-DTA (Ours)	0.128	0.906	0.798

**Table 4:** Ablation study on the Davis dataset. Arrows indicate the direction of optimal performance (↓: lower is better; ↑: higher is better).

Configuration	MSE ↓	CI ↑	$r_{m}^{2}$↑
w/o ASAG (global pooling)	0.215	0.903	0.712
w/o Adaptive (SAGPooling, k=0.5)	0.198	0.909	0.741
w/o Drug Seq	0.208	0.904	0.722
w/o Protein Seq	0.202	0.906	0.735
w/o Fingerprint	0.195	0.910	0.750
ASAG-DTA (Full)	0.186	0.915	0.765

**Table 5:** Ablation experiments on the Davis dataset (DynHeter-DTA) (reproduced under identical experimental settings as our prior work [36] for fair comparison).

Model	MSE	CI	$r_{m}^{2}$
Without a heterogeneous graph, using GCN	0.257	0.885	0.660
Without a heterogeneous graph, using GIN and SAGPooling	0.229	0.892	0.695
Dynamic heterogeneous graph with GIN and SAGPooling	0.130	0.923	0.828

**Table 6:** Ablation experiments on KIBA dataset (DynHeter-DTA) (reproduced under identical experimental settings as our prior work [36] for fair comparison).

Model	MSE	CI	$r_{m}^{2}$
Without heterogeneous graph, using GCN	0.141	0.861	0.765
Without heterogeneous graph, using GIN and SAGPooling	0.135	0.890	0.801
Using dynamic heterogeneous graph with GIN and SAGPooling	0.123	0.908	0.821

**Table 7:** Cold-start evaluation on the Davis dataset (MSE/CI).

Setting	Random Split	Cold Drug	Cold Protein	Cold Pair
DGraphDTA	0.202/0.904	0.245/0.875	0.238/0.879	0.312/0.832
ASAG-DTA (Ours)	0.186/0.915	0.221/0.889	0.215/0.892	0.289/0.851

**Table 8:** Performance on the Davis dataset (mean ± std over 5 runs). Arrows indicate the direction of optimal performance (↓: lower is better; ↑: higher is better).

Model	MSE ↓	CI ↑	$r_{m}^{2}$↑
DGraphDTA	0.202 ± 0.008	0.904 ± 0.003	0.700 ± 0.012
ASAG-DTA	0.186 ± 0.006	0.915 ± 0.002	0.765 ± 0.009
*p*-value	p<0.01	p<0.01	p<0.01

**Table 9:** Hyperparameters for ASAG-DTA.

Parameter	Value
Epochs	2000
Batch size	512
Optimizer	Adam
Learning rate	0.001
Dropout (heterogeneous/drug/protein)	0.2/0.3/0.5
Embedding dimensions	256/1024/128
FC layers	1024 → 512 → 1
ASAGPooling initial ratio	0.5 (α=0)
Transformer layers	4
Transformer attention heads	4
Transformer FFN dimension	512

**Table 10:** Sensitivity to learning rate on the Davis dataset. Arrows indicate the direction of optimal performance (↓: lower is better; ↑: higher is better).

Learning Rate	MSE ↓	CI ↑	$r_{m}^{2}$↑
$1\times 10^{-4}$	0.201	0.908	0.742
$5\times 10^{-4}$	0.191	0.912	0.756
$1\times 10^{-3}$	0.186	0.915	0.765
$2\times 10^{-3}$	0.189	0.913	0.759
$5\times 10^{-3}$	0.198	0.907	0.741

**Table 11:** Sensitivity to dropout configuration on the Davis dataset. Arrows indicate the direction of optimal performance (↓: lower is better; ↑: higher is better).

Dropout (Drug/Protein/Het)	MSE ↓	CI ↑	$r_{m}^{2}$↑
0.1/0.2/0.1	0.192	0.911	0.753
0.2/0.3/0.2	0.188	0.913	0.760
0.3/0.5/0.2	0.186	0.915	0.765
0.4/0.6/0.3	0.190	0.912	0.757
0.5/0.7/0.4	0.195	0.908	0.748

**Table 12:** Sensitivity to embedding dimension on the Davis dataset. Arrows indicate the direction of optimal performance (↓: lower is better; ↑: higher is better).

Embedding Dimension	MSE ↓	CI ↑	$r_{m}^{2}$↑	Params (M)
64	0.201	0.906	0.740	1.8
128	0.194	0.910	0.752	2.0
256	0.186	0.915	0.765	2.2
384	0.185	0.915	0.766	2.6
512	0.185	0.916	0.767	3.1

**Table 13:** Sensitivity to Transformer layers on the Davis dataset. Arrows indicate the direction of optimal performance (↓: lower is better; ↑: higher is better).

Transformer Layers	MSE ↓	CI ↑	$r_{m}^{2}$↑	Training Time (h)
2	0.195	0.909	0.751	4.1
3	0.190	0.912	0.758	4.8
4	0.186	0.915	0.765	5.2
5	0.188	0.914	0.762	5.9
6	0.191	0.911	0.755	6.5

**Table 14:** Sensitivity to ASAGPooling initial ratio on the Davis dataset.

Initial Ratio	Final MSE	Final CI	Final $r_{m}^{2}$	Converged Ratio
0.3	0.187	0.914	0.764	0.46
0.5	0.186	0.915	0.765	0.48
0.7	0.187	0.914	0.764	0.47

**Table 15:** Computational comparison on the Davis dataset.

Configuration	Training Time (h)	Inference Time (ms/sample)	Parameters
Global pooling	4.2	2.3	2.1 M
TopKPooling (k=0.5)	4.8	2.8	2.2 M
SAGPooling (k=0.5)	5.1	3.0	2.2 M
ASAGPooling (Ours)	5.2	3.1	2.2 M + 2

**Table 16:** Description of atomic features in the drug molecular graph.

Atomic Feature	Dimension
Atom symbol	44
Atom degree	1
Total number of hydrogen atoms	1
Number of implicit hydrogen atoms in the atom	1
Whether the atom is aromatic	1
Total	78

**Table 17:** Residue features in the protein graph.

Feature	Dimension
Residue symbol	21
Special position score matrix	21
Whether it is aliphatic	1
Whether it is aromatic	1
Whether it is polar neutral	1
Whether it is an acidic charge	1
Whether it is an alkaline charge	1
Residue weight	1
Negative logarithm of -COOH dissociation constant	1
Negative logarithm of -NH_2_ dissociation constant	1
Negative logarithm of dissociation constants for other groups	1
pH at isoelectric point	1
Hydrophobicity at pH = 2	1
Hydrophobicity at pH = 7	1
Total	54

**Table 18:** Overlap between ASAGPooling-retained residues and ligand-binding pockets.

Complex	PDB ID	Total Residues	Binding Pocket Size	Retained/Binding (%)
Dasatinib-SRC	2SRC	386	42	82.3%
Imatinib-ABL1	1IEP	488	38	78.9%
Erlotinib-EGFR	1M17	590	45	84.5%
Average				81.9%

## Data Availability

The Davis and KIBA datasets are publicly available. Our code and processed data will be released upon publication.
